# Feasibility and Acceptability of SARS-CoV-2 Antigen Rapid Diagnostic Testing in High-Risk Markets and Trade Hubs in Kampala, Uganda

**DOI:** 10.4269/ajtmh.23-0899

**Published:** 2025-02-25

**Authors:** Isaac Ssewanyana, Sam Acellam, Martha Ampumuza, Hellen Nansumba, Susan Nabadda, Grace Kushemererwa, Victor Bigira, Sarah Zalwango, Chris Oundo, Richard Walyomo, Isaiah Chebrot, Dennis Mike Buluma, Alex Ndyabakira, Pallavi Dani, Anne Hoppe

**Affiliations:** ^1^ Central Public Health Laboratory, Ministry of Health, Kampala, Uganda;; ^2^ Kampala Capital City Authority, Kampala, Uganda;; ^3^ FIND, Geneva, Switzerland;; ^4^ Elizabeth Glaser Pediatric AIDS Foundation, Geneva, Switzerland

## Abstract

Congregate settings are high-risk places for severe acute respiratory syndrome coronavirus 2 (SARS-CoV-2) transmission, making strategies that rely solely on hospital-based testing ineffective in curbing transmissions. We therefore evaluated the feasibility, utility, and acceptability of testing with SARS-CoV-2 antigen rapid diagnostic tests (Ag-RDTs) in markets and trade hubs in Kampala, Uganda. Between June and September 2022, we conducted a prospective operational research study in five divisions of Kampala. Four rounds of monthly cross-sectional surveys were conducted at one market and one trading hub per division, resulting in a total of 13,086 volunteers tested. Females were more likely than males to be tested (54% versus 46%), which aligns with sex-based differences in health-seeking behavior. More tests were conducted in markets (68%) compared with trade centers (32%). Several interventions increased overall demand for testing, including 1) awareness campaigns and mobilization activities; 2) the movement of teams across congregate settings; 3) the optimization of workflow; and 4) testing traders at their workstations. The overall positivity rate during the 4 months was 0.6% (78/13,086). There was a steady decline in positivity rates by month, aligning with the trend observed at the national level. Of the 78 positive index cases identified, 105 contacts were traced; 71% of these could be reached. None of the positive patients successfully self-isolated for the 14 days specified in national guidelines. Nevertheless, this study demonstrates that testing market dwellers with Ag-RDTs is not only acceptable and feasible in Uganda but also an important public health tool for the timely detection of SARS-CoV-2. This approach may be replicated in similar settings.

## INTRODUCTION

Since its initial identification in Wuhan, China, in November 2019, severe acute respiratory syndrome coronavirus 2 (SARS-CoV-2) has resulted in a global pandemic with more than 630 million infections and 6.5 million deaths formally reported.^1^ The pandemic manifested in multiple waves of infections in different countries, including Uganda.^2^ Like elsewhere, the government of Uganda imposed strict national lockdowns, during which most businesses and social services remained closed for long periods, ranging from 3 to 6 months. Only essential services, such as health, security, communication, and food markets, remained open while observing coronavirus disease 2019 (COVID-19) standard operating procedures.^3^ Although the lockdown measures slowed viral transmission, these prolonged measures also negatively impacted the local economy, people’s quality of life, and their livelihoods. The lockdown led to the widespread loss of income and depletion of business capital and personal savings. These economic disruptions are estimated to continue affecting Uganda and its development performance until 2030.^4^

Kampala is Uganda’s capital and, as such, a major business center contributing more than 60% of the country’s gross domestic product. The city is the industrial hub and serves as a significant marketplace for agricultural and imported goods. It is estimated that a total of 3.6 million people congregate in Kampala’s markets and trade hubs and disperse to the rest of the countryside daily.^5^ Kampala traders are in constant close contact with a large, dynamic population, which puts them at potentially high risk of contracting and transmitting SARS-CoV-2.

Throughout the pandemic, the national COVID-19 response was primarily reactive to symptomatic suspected cases, which is a lagging indicator of early detection of community-based outbreaks. The availability of antigen rapid diagnostic tests (Ag-RDTs) enabled screening for both symptomatic and asymptomatic cases in community settings, facilitating the detection of SARS-CoV-2 transmission and guiding rapid responses to control it.

This operational research project assessed the feasibility and acceptability of routine SARS-CoV-2 Ag-RDT screening for early detection and control of transmission in markets and trade hubs in Kampala. Controlling infections in Kampala has far-reaching benefits for managing SARS-CoV-2 transmission throughout the rest of the country.

## MATERIALS AND METHODS

We conducted a prospective operational research study in five divisions of the Kampala Capital City Authority (KCCA), namely, Nakawa Division, Makindye Division, Central Division, Kawempe Division, and Lubaga Division. Two rounds of cross-sectional surveys were conducted at one trade hub per division during June and July 2022 (five sites), and four rounds of testing surveys were conducted at one market per division from June to September 2022 (five sites).

**Figure 1.**
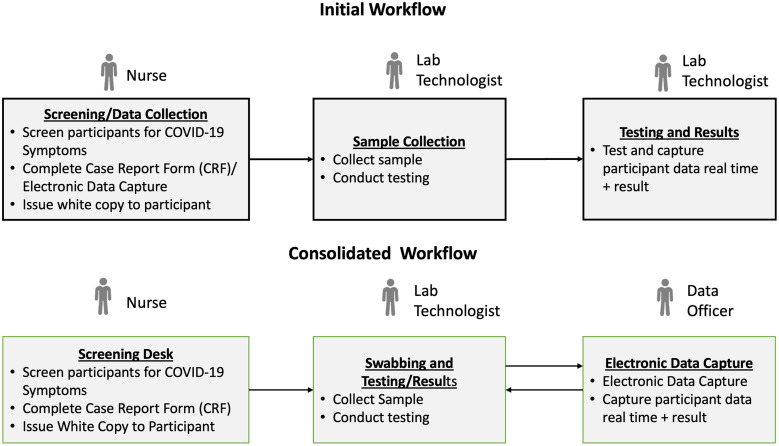
Workflow diagram for antigen rapid diagnostic testing rounds. This figure depicts the operational flow during round 1, known as the initial workflow, and the adapted process applied in rounds 2–4, termed the consolidated workflow. It outlines the sequence of testing activities, from participant recruitment through to data collection and follow-up, demonstrating the evolution of our testing strategy over the study period.

Testing was conducted at temporary testing centers, which were divided into data collection, sample collection, and testing sections. In the first round, all relevant data was collected before testing; however, the workflow was revised for subsequent rounds, allowing data to be collected while participants waited for their results (Figure 1).

**Figure 2.**
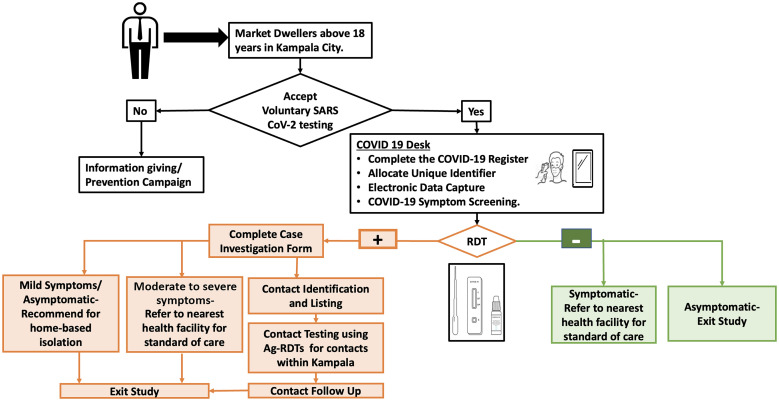
Flowchart diagram for coronavirus disease 2019 testing in markets and trading hubs.

All market and trading hub dwellers aged 18 years and above, who were willing to participate in voluntary Ag-RDT testing within Kampala City, were enrolled in the study. The participants included suppliers, market stall operators, and buyers. Those who were tested were classified as suppliers, market stall or shop operators, and buyers (Figure 2).

The details of individuals who wished to be tested were captured using the Ministry of Health’s (MoH’s) laboratory investigation form for COVID-19 before their samples were collected and tested using Ag-RDTs. Test results were returned to the participants within 15–30 minutes of sample collection. All positive participants were advised to self-isolate at home for 14 days, in accordance with national guidelines.^6^ Participants presenting with moderate to severe signs and symptoms were referred to a nearby hospital for further clinical management. Because of the limited number of voluntary market dwellers presenting at these testing points, this approach was complemented by a pragmatic strategy involving KCCA surveillance officers, who approached traders and tested those interested in being tested at their stalls.

For tracing the contacts of positive cases, the MoH’s contact tracing and surveillance tools were adopted. When a case was identified, the case details were shared with the KCCA rapid response surveillance team. A KCCA surveillance officer interviewed the patient to identify and enlist all possible contacts from the preceding 14 days. Additional information was obtained from individuals in the vicinity of the patient. Contacts were informed, and when possible, physically located; specimens were collected and tested using Ag-RDTs. Results were provided within 20–30 minutes after sample collection. All contacts were advised on prevention measures, and those who tested positive were advised to self-isolate for a period of 14 days in accordance with national guidelines.^6^ Follow-up phone calls were made to all positive participants and their contacts to assess adherence to self-isolation.

The KCCA divisions’ rapid response surveillance teams conducted contact tracing in all five divisions of Kampala. Contacts of participants reported to reside outside Kampala were not traced; however, KCCA reported these individuals to the COVID-19 response teams in the respective districts for follow-up.

### Data collection, storage, and analysis.

We adopted the MoH COVID-19 standard tools, including the laboratory investigation form, contact listing form, and contact follow-up forms, for the purpose of this study to facilitate linkage with national systems. The laboratory investigation form for COVID-19 captured personal identification, contact, and surveillance and epidemiological data. Participant data included demographics, clinical information, laboratory testing information, and details of community exposures. All Ag-RDT test results were captured using this tool. Using the Android-based version of the laboratory investigation form (ELIF app, Uganda National Health Laboratory Services, Kampala, Uganda), data were collected and uploaded into the centralized database and dashboard in real-time. All data captured using the ELIF application were uploaded into the national COVID-19 surveillance database to contribute to routine national reporting.

The study analysis was performed using the study database. Data for analysis were downloaded in the form of Microsoft Excel sheets (.xls; Microsoft Corp, Redmond, WA) from the Results Dispatch System.

## RESULTS

### Proportion of people who accepted voluntary Ag-RDT testing.

Between June and July 2022, testing was offered at both markets and trade hubs (10 sites) within each of the five divisions of Kampala. In the subsequent 2 months, testing was offered at the five markets only (one per division). The average footfall at these testing sites is estimated to be at least 5,300 per site per day.^7^ Each site aimed to perform 300 tests per round across four rounds, totaling 1,200 tests per site. With 10 sites, this would typically result in 12,000 tests. Additionally, for each round, we estimated contact testing for 45 positive participants per site, which, over four rounds, added an additional 1,800 tests across all sites. Thus, it was estimated that at least 13,800 people could be reached via this strategy over a 4-month period.

A total of 13,086 out of 13,800 participants (95%) were tested. Participants were equally distributed among the five divisions (Table 1), implying similar levels of testing acceptability across Kampala. More female (54%) than male (46%) adults participated in the voluntary testing, reflecting trends observed in sex-based differences in health-seeking behavior.

**Table 1:** Demographic characteristics of the 13,086 participants enrolled in this study

Characteristic		*N* = 13,086
Median age, years (IQR)		30 (16)
Sex,* *n* (%)	Male	6,018 (46)
	Female	7,063 (54)
Division, *n* (%)	Central	2,779 (21)
	Makindye	2,786 (21)
	Kawempe	2,447 (19)
	Lubaga	2,360 (18)
	Nakawa	2,714 (21)

IQR = interquartile range.

* Five missing data.

During the first 2 months of the study, four times more tests were conducted in markets (80%) compared with trade centers (20%), leading to the cessation of testing in trade centers for the final 2 months of implementation. The variation in access to testing services was likely due to differences in mobilization activities and accessibility. In the markets, KCCA’s rapid response surveillance teams conducted COVID-19 awareness campaigns through the local community and traders’ leadership. Our study team leveraged this platform to promote the study and encourage market dwellers to participate in voluntary testing. Open market stalls were also more accessible to testing teams.

**Figure 3.**
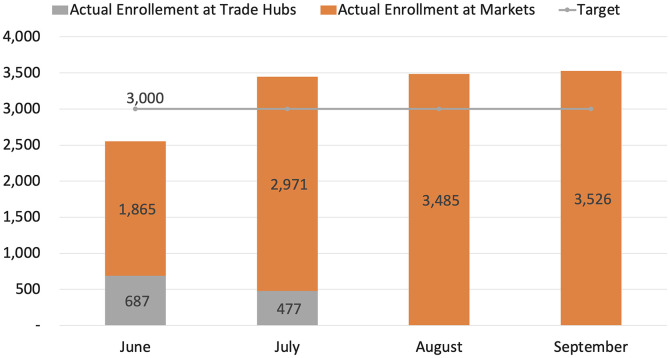
Targets versus actual enrollments between June and September 2022 for index cases.

**Figure 4.**
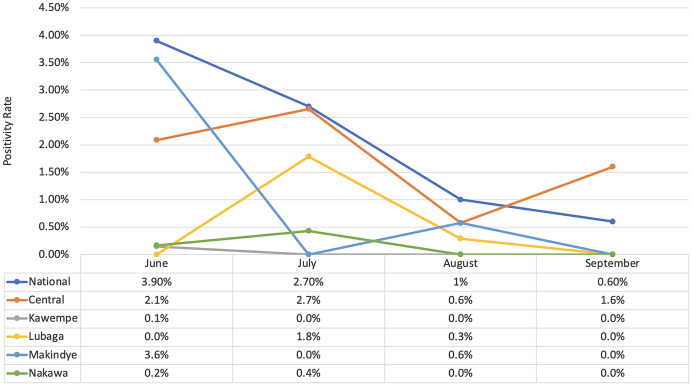
Overall positivity rate over time.

The total number of tests conducted was slightly lower than the target (Figure 3), primarily due to low testing demand in the first round and the cessation of activities in trade hubs during the final two rounds. The lessons learned in the first round led to interventions that resulted in demand exceeding targets in the markets during rounds 2–4 (Figure 3). The interventions that increased demand included 1) more awareness campaigns and mobilization activities, 2) the movement of teams across congregate settings in the markets, 3) the optimization of workflow (Figure 1), and 4) testing traders at their workstations (stalls or shops).

Awareness campaigns and mobilization activities that utilized megaphones, peer leaders, and community health workers or village health teams helped alert residents to the availability of free testing and addressed misconceptions about COVID-19. Additionally, revising the workflow reduced waiting times by 10 to 15 minutes, and approaching traders at their workstations was beneficial to them because it prevented loss of time and income during testing. However, despite these interventions, very few buyers and suppliers were willing to get tested.

### Antigen rapid diagnostic test positivity rate.

The overall positivity rate during the 4 months of cross-sectional surveys was 0.6% (78/13,086) with the central division contributing the highest number of positive cases (47, 0.4%) and both Makindye and Nakawa registering no cases. There was a steady decline in positivity rates month on month (1.2%; 1%; 0.3%; 0.3%) from June to September, and this trend was also observed at the national level (3.9%; 2.7%; 1.0%; 0.6%; Figure 4). The national positivity rate was overall higher than the study’s positivity rate. This difference can be attributed to positivity enrichment at the national level due to testing symptomatic suspects (mostly in hospital settings), compared with primarily healthy business communities in the study.

### Contact tracing and self-isolation of positive cases.

Out of the 78 positive index cases identified, 105 contacts were traced, and 71% of these were reachable. Among the contacts, the Kawempe Division recorded the highest overall positivity rate (2.7%); however, the number of positive cases is too small to draw any meaningful conclusions (Table 2).

**Table 2:** Positivity data on index and contact cases across all five divisions

Division	Overall Positivity, *N* (%)	Index Case Positivity, *N* (%)	Total Number of Contacts Identified	Total Number (%) of Contacts Tested	Contact Case Positivity, *N* (%)
Central	47 (0.4)	47 (0.4)	56	47 (72)	0 (0.0)
Kawempe	16 (0.1)	14 (0.1)	20	22 (73)	2 (2.7)
Lubaga	13 (0.1)	9 (0.1)	16	4 (16)	1 (1.3)
Makindye	1 (0.0)	1 (0.0)	6	0 (0)	0 (0.0)
Nakawa	4 (0.0)	4 (0.0)	7	2 (13)	0 (0.0)

Several contacts were found to be residing outside of Kampala, which was beyond our area of jurisdiction, and there was a reluctance among the index case participants to volunteer information. Only 75 contacts residing within Kampala were tested, and three positives were identified (two in Kawempe and one in Lubaga).

There were no positive participants who successfully self-isolated for the required 14 days in accordance with the national guidelines.

## DISCUSSION

Our findings underscore the feasibility and acceptability of conducting community-based testing using Ag-RDTs as a public health tool for the timely detection of SARS-CoV-2, facilitating rapid response and containment efforts. Testing in markets was more successful than testing in trade hubs because it allowed for traders to be tested at their workstations and made it easier to conduct robust mobilization and awareness campaigns. These campaigns not only provided accurate information to dispel COVID-19-related misconceptions and stigma but also integrated relevant health messaging, as demonstrated by the inclusion of Ebola outbreak information alongside our SARS-CoV-2 testing efforts.^8^

Our testing acceptance rates align with, or exceed, those reported in other settings, underscoring the potential for replicating this model in similar urban settings globally. Although we did not systematically collect data on the reasons for declining SARS-CoV-2 testing, the stigma associated with COVID-19 and the fear of loss of income during quarantine and isolation were identified as key barriers to testing. Future studies may benefit from structured surveys or interviews to quantitatively assess these aspects because addressing barriers is crucial and integral to the design of future testing strategies to ensure higher uptake and public cooperation. Moreover, expanding contact tracing beyond Kampala is necessary to prevent regional transmission.

The convergence of our SARS-CoV-2 testing rounds with the onset of the Ebola outbreak in Uganda presented unique challenges while also offering valuable insights into the management of simultaneous public health crises. This situation highlights the need for adaptable, multifaceted public health strategies capable of responding to the dynamic landscape of infectious diseases. As the global community continues to face emerging and reemerging infectious threats, the lessons learned from this study are critical for informing scalable and sustainable health responses, ensuring preparedness, and safeguarding public health.
